# Reduction of Radiometric Miscalibration—Applications to Pushbroom Sensors

**DOI:** 10.3390/s110606370

**Published:** 2011-06-16

**Authors:** Christian Rogaß, Daniel Spengler, Mathias Bochow, Karl Segl, Angela Lausch, Daniel Doktor, Sigrid Roessner, Robert Behling, Hans-Ulrich Wetzel, Hermann Kaufmann

**Affiliations:** 1 Section 1.4 Remote Sensing, Helmholtz Centre Potsdam–GFZ German Research Centre for Geosciences, Telegrafenberg, 14473 Potsdam, Germany; E-Mails: daniel.spengler@gfz-potsdam.de (D.S.); mathias.bochow@gfz-potsdam.de (M.B.); karl.segl@gfz-potsdam.de (K.S.); sigrid.roessner@gfz-potsdam.de (S.R.); robert.behling@gfz-potsdam.de (R.B.); wetz@gfz-potsdam.de (H.-U.W.); hermann.kaufmann@gfz-potsdam.de (H.K.); 2 Department of Landscape Ecology, Helmholtz Centre for Environmental Research–UFZ, Permoserstr.15, 04318 Leipzig, Germany; E-Mails: angela.lausch@ufz.de (A.L.); daniel.doktor@ufz.de (D.D.)

**Keywords:** radiometric, correction, miscalibration, stripes, nonlinearity, hyperspectral, AISA, Hyperion, EnMAP, MoLaWa, PROGRESS

## Abstract

The analysis of hyperspectral images is an important task in Remote Sensing. Foregoing radiometric calibration results in the assignment of incident electromagnetic radiation to digital numbers and reduces the striping caused by slightly different responses of the pixel detectors. However, due to uncertainties in the calibration some striping remains. This publication presents a new reduction framework that efficiently reduces linear and nonlinear miscalibrations by an image-driven, radiometric recalibration and rescaling. The proposed framework—Reduction Of Miscalibration Effects (ROME)—considering spectral and spatial probability distributions, is constrained by specific minimisation and maximisation principles and incorporates image processing techniques such as Minkowski metrics and convolution. To objectively evaluate the performance of the new approach, the technique was applied to a variety of commonly used image examples and to one simulated and miscalibrated EnMAP (Environmental Mapping and Analysis Program) scene. Other examples consist of miscalibrated AISA/Eagle VNIR (Visible and Near Infrared) and Hawk SWIR (Short Wave Infrared) scenes of rural areas of the region Fichtwald in Germany and Hyperion scenes of the Jalal-Abad district in Southern Kyrgyzstan. Recovery rates of approximately 97% for linear and approximately 94% for nonlinear miscalibrated data were achieved, clearly demonstrating the benefits of the new approach and its potential for broad applicability to miscalibrated pushbroom sensor data.

## 1. Introduction

The potential of imaging spectroscopy to provide more and better information about the Earth than do multispectral instruments is currently accompanied by an intensified development and availability of new hyperspectral airborne and spaceborne sensors. The new generation of hyperspectral sensors utilise the pushbroom technology, enabling an integration time per detector element. Therefore, these sensors obtain a better Signal-to-Noise Ratio (SNR) compared to whiskbroom scanners. However, the use of detector arrays in the sensor design requires a more precise radiometric calibration. Even small variations will cause striping effects in the image data that aggravate subsequent analyses such as classification and segmentation [1], and these effects should be reduced by performing radiometric rescaling beforehand. Miscalibration can be divided into two basic types—additive (offset) and multiplicative (slope) degradation—and can be perceived visually as image stripes. Offsets are used to incorporate detector-dependent dark current, which is caused by thermally generated electrons [2]. In contrast, slopes are used to directly assign radiance to DN. Hence, striping reduction should suppress stripes and at the same time preserve the spectral characteristics of the imaged surface materials. In the literature, specific approaches for destriping of slope stripes, offset stripes or both exist, and these are based primarily on methods such as interpolation [3,4], local or global image moments [1,5–7], filtering [8–11] or complex image statistics of log transformed slopes [12–14]. However, a replacement of original, but miscalibrated radiances should be applied only if information is completely missing or erroneous. In this work, a framework is presented that reduces linear and nonlinear stripes and preserves spectral characteristics by radiometric rescaling. This framework, Reduction Of Miscalibration Effects (ROME), consists of a linear and a nonlinear slope reduction as well as an offset reduction, which are performed consecutively and evaluated by specific image quality metrics, such as the Signal-to-Noise-Ratio (SNR). The slope reduction is performed for each detector element and band without any information from other detector elements. In case dark current related differences between adjacent detectors have not been balanced by a foregoing calibration, they need to be reduced. For this purpose an offset reduction was developed that performs in a moving window and incorporates image statistics of adjacent image columns. Both basic reduction steps incorporate spatial and spectral probability distributions and integrate striping related redundancies. Subsequent to the degradation reduction, a radiometric rescaling is proposed.

The rescaling aims to adjust the radiometric scale by considering areas of lowest reduction. This is necessary since uncertainties remain in the estimation of parameters (e.g., detector resolution in the linear slope reduction) and in the incorporation of miscalibrated reference areas (e.g., potential miscalibration of the first image column as reference for the offset reduction).

Additionally, it will be shown how potential trends or frequency undershoots caused by corrections themselves or by low SNR can be suppressed. In addition, an adapted data dimensionality reduction is proposed, which desensitises striping reduction approaches in the presence of edges and increases computational speed. Here, Minkowski metrics, gradient operators and edge extraction algorithms are combined to exclude discontinuities such as edges and impulse noise from further analyses if they do not dominate the image content [15–17]. To study the impacts of different linear miscalibrations on the performance of the proposed method, a specific set of grey valued images was randomly striped by linearly varying the slope and/or offset. The nonlinear correction facilities were tested by destriping a simulated EnMAP (Environmental Mapping and Analysis Program) scene [18–20], which was not corrected for nonlinear effects. In addition, a set of hyperspectral, miscalibrated AISA Dual [21] and Hyperion scenes [22,23] were processed.

## 2. Materials

Four grey valued images from the image database of the Signal and Image Processing Institute (SIPI) of the University of California [24], 512 × 512 pixels in size, and six hyperspectral scenes were selected to evaluate the performance of the proposed miscalibration reduction. In the following, the x-dimension is considered as column or across track, the y-dimension is considered as row or along track, the spectral dimension is considered as band and single banded images or one band of a multi banded image are considered as image.

### 2.1. Grey Valued Image Samples

**Figure 1.**
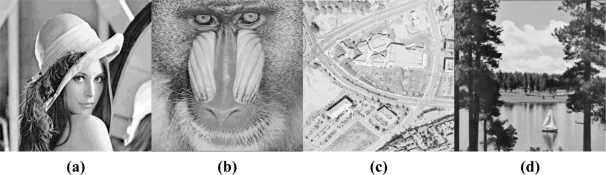
Grey-scaled representations [24] of (**a**) ‘Lenna’, (**b**) ‘Mandrill’, (**c**) ‘Aerial’ and (**d**) ‘Sailboat on lake’.

To simulate different types of linear miscalibration, each of the four grey valued images from the SIPI image database (Figure 1) were artificially degraded 400 times by linear multiplicative and/or additive Gaussian white noise [25].

For every specific noise degradation level and type (slope, offset) the Gaussian white noise was randomly generated [25] and standardised to provide a mean equal to zero and a standard deviation equal to one. The noise degradation was performed 1600 times to achieve a statistical variety. In order to simulate a specific noise level out of 80 predefined noise levels, the white noise was rescaled by a linear transformation to a defined minimum and maximum. This resulted in generation of 1600 different sets of Gaussian white noise comprising of 20 different noise sets for the 80 different noise levels.

Each set was applied to each grey valued image and consists of 5 variations representing the different noise types slope and offset, slope only, offset only, slope and a priori knowledge and offset and a priori knowledge. In the result each grey valued image was 400 times differently degraded.

The rescaled multiplicative noise ranged in maxima from 1 to 1789 (mult1), whereas the minimum was fixed to 0.0001 (mult2) to enable an impact of the multiplicative degradation. The rescaled additive noise ranged in maxima from 5.59 to 10,000 (off1) and in minima from −5.59 to −10,000 (off2). The rescaling was based on exponential functions to simulate small and large degradations. To detect potential scaling effects, the scaling was varied four times within the 80 different noise levels:

Case 1: multiplicative from mult2 to mult1 and additive from mult2 to reversed off1.
Case 2: multiplicative from mult2 to mult1 and additive from mult2 to off1.
Case 3: multiplicative from mult2 to mult1 and additive from reversed off2 to reversed off1.
Case 4: multiplicative from mult2 to mult1 and additive from off2 to off1.

### 2.2. Hyperspectral Image Samples

A set of six specific miscalibrated hyperspectral images were additionally destriped to test the proposed approach on images that were acquired either from aircraft or from satellite and degraded by either linear or nonlinear miscalibrations. For this purpose, three hyperspectral AISA DUAL scenes [21], two Hyperion scenes [22,23] and one EnMAP scene [18–20] were selected. The specific properties of these scenes and the reasons for their selection are described below.

**Figure 2.**
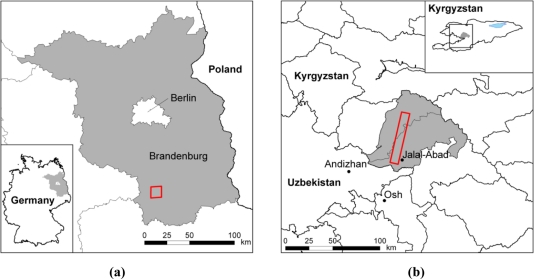
Study regions: (**a**) ‘Fichtwald’ (AISA DUAL) and (**b**) ‘Kara-Bulak’ (Hyperion).

The three AISA DUAL scenes were acquired on September 23rd 2010 between 1 p.m. and 3 p.m. for the ‘Fichtwald’ study region in Eastern Germany (Figure 2a). These data will be used in the Monitoring of Landscape Water Balance (MoLaWa) project.

The AISA DUAL system consists of two separate pushbroom sensors, AISA Eagle (400–970 nm) and AISA Hawk (970–2450 nm), which are mounted on a stabilised aircraft platform [21]. According to a mean flight height of 1620 m above ground, a spatial resolution of 2 m was achieved. Acquired data had a varying spectral resolution of approximately 2.3 nm for the Eagle sensor and approximately 6.3 nm for the Hawk sensor. All datasets exhibit visually perceivable striping patterns, appearing to indicate sensor miscalibrations.

The two hyperspectral Hyperion image scenes [22,23] of almost identical spatial coverage of approximately 7.7 km × 90 km were acquired on the 14th and 22nd of June 2010 from a sun-synchronous 705-km-high orbit with a spatial resolution of 30 m. They partly cover a study region in the Southern Tian Shan Mountains along the Eastern rim of the Fergana Basin in Kyrgyzstan (Figure 2b). They will be analysed for lithological investigations [26–28] within the framework of the Potsdam Research Cluster for Georisk Analysis, Environmental Change and Sustainability (PROGRESS).

Due to SNR considerations, only 198 bands are routinely processed for generating level 1 images [22]. Both data takes are affected by vertical striping in all spectral bands, which may indicate sensor miscalibrations similar to that of the AISA DUAL scenes. To perform further processing and subsequent automated information extraction, these stripes must be removed. Missing data, for example, those in Hyperion scenes, were bidirectional interpolated by Piecewise Cubic Hermite Polynomials (PCHIP) similar to Tsai *et al*. [4].

The approach was tested further by using data generated to be like those from future EnMAP sensor [19]. EnMAP is a German-built hyperspectral pushbroom space sensor scheduled for launch in 2015. It will measure in the 420–2450 nm spectral range using 244 bands at a varying spectral sampling of 6.5–10 nm. Images will cover 30 × 30 km at an approximate ground sampling distance of 30 m. It also includes different inflight-calibration means such as a solar diffuser, a main sphere for radiometric stability measurements, a small sphere for spectral calibration and FPA LEDs for detector non-linearity calibration. An EnMAP scene simulator has been developed at the GFZ Potsdam that is able to generate realistic EnMAP-like data in an automatic way, applying a set of user-driven instrumental, atmosphere and scene parameters [18,20].

**Figure 3.**
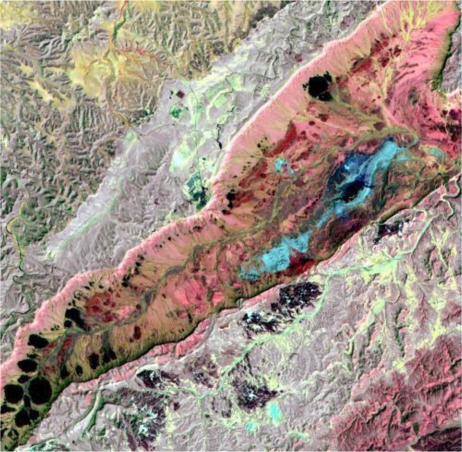
False coloured RGB image [band 12–479 nm (blue), 65–801 nm (green) and 213–2201 nm (red)] of the ‘Makhtesh Ramon’ study region as a simulated EnMAP scene.

This simulator is used for the optimisation of instrument specifications and the development and validation of data processing and calibration algorithms. An example of a simulated EnMAP image is depicted in Figure 3 showing the Makhtesh Ramon in Israel. This location in the southern Israeli Negev Desert is one of the most promising sites worldwide for hyperspectral sensor calibration. The image processing requires high spectral and spatial resolution data as input, simulated by merging Spot-5 panchromatic and multispectral data with representative endmember field spectra. For this investigation, it was assumed that the detector non-linearity calibration indicates that is not operative. This means that the simulated L1-process fails to correct for non-linearity. As a result, fine nonlinear striping patterns remain visible in the image data.

## 3. Methods

### 3.1. Problem Definition

Different physical detector characteristics of a pushbroom sensor produce image stripes in acquired raw data. These stripes are then corrected by radiometric in-flight, vicarious [29,30], flat field [31] or laboratory calibrations that transform raw data to radiance. Any remaining stripes are, therefore, caused by miscalibrations. Considering one detector element of a pushbroom sensor, the signal S can be approximated by a nonlinear relation [32,33] to:

(1) $$\text{S}({\text{e}}^{-})\propto \frac{\text{F}\cdot \text{L}\cdot \text{A}\cdot {\text{tan}}^{2}(\frac{\text{FOV}}{2})\cdot \tau \cdot \text{T}\cdot \lambda \cdot \eta \cdot \text{SSI}}{\text{h}\cdot \text{c}\cdot {\text{n}}_{{\text{e}}^{-}}^{2}}$$

where L is the at-sensor-radiance, A is the aperture of the sensing instrument, FOV is the field of view, T is the integration time, SSI is the Spectral Sampling Interval in respect to the Full Width at Half Maxima, h is the Planck constant, c is the speed of light, n_e_^−^ is the number of collected electrons, τ is the optical transmission, λ is the centre wavelength, is the quantum efficiency and F is the filter efficiency. However, the detector signal must be related to a recordable and transmittable digital number (DN), which may be given by the following equation:

(2) $$\text{DN}=\frac{(\text{S}+\text{N})\cdot {\text{DN}}_{\text{max}}}{\text{FWC}}+{\text{DN}}_{0}\wedge \text{S}\leq \text{FWC}$$

where N is a noise term incorporating Shot-Noise, read-out noise and dark noise, DN_max_ is the radiometric resolution, FWC is the Full Well Capacity that defines detector saturation, and DN_0_ is the dark current. Subsequent laboratory measurements are then used to estimate transformation parameters, either for the transformation of at-sensor radiance L to digital number DN considered to be radiometric calibration or, *vice versa*, considered to be radiometric scaling [34].

Calibration measurements are performed by sensing known physical targets and by a subsequent evaluation of the sensing results. The association is often realised by a polynomial least squares fit, which minimises the differences between modelled and measured at-sensor radiance [35,36]. This minimisation of the merit function can then be used to obtain the coefficients for radiometric transformations:

(3) $$\chi^{2}=\Sigma_{\text{j}=1}^{{\text{N}}_{\text{targets}}}{\left[\text{L}-({\text{c}}_{0}+\Sigma_{\text{i}=1}^{\text{M}}\;{\text{c}}_{\text{i}}\cdot {\text{DN}}^{\text{i}})\right]}^{2}\wedge \text{M}\geq 1;\;{\text{N}}_{\text{targets}}\geq 2$$

where N_targets_ denotes the number of calibration targets, c_0_ is the offset contrary to the dark current, and M is the polynomial degree. In many cases, the assignment is performed linearly, that is, M becomes 1. It is not possible or practical to calibrate a sensor for each image acquisition, which results in relying on calibration parameters that may no longer be up-to-date. This can lead to vertical stripes requiring a new calibration based solely on image data. Additionally, it is necessary to assess the stripe type to perform the right reduction—multiplicative or additive—linear or nonlinear. This can be achieved by an inspection of the outputs of different reduction approaches, either manually or automatically, which may be more reliable if the differences are small.

### 3.2. Assessment of Stripe Type and Masking of Discontinuities

Striping reduction can be divided into two types—an additive c_0_ and a multiplicative c_1..M_ reduction. Because how an image is degraded is unknown, all c_0_, c_1_ and c_2..M_ reductions should be performed and evaluated. The evaluation can be based on the assessment of the *a priori* and the *a posteri* Signal-To-Noise Ratio (SNR) [37,38]. The SNR is determined for each band as the ratio between the global mean and the local standard deviation representing the highest probability of all local standard deviations resulting from a moving window approach [38]. The ratio of the *a posteri* SNR and the *a priori* SNR, considered to be the change in the SNR (cSNR), may then be used as reduction-quality indicator. Because only SNR relations are incorporated, the impacts of different land cover types on the SNR assessment are also suppressed [39]. Hence, a cSNR less than one indicates that the preceding reduction caused degradation, which must be revoked and *vice versa*.

Discontinuities such as edges and impulse noise have a specific impact on the assessment of stripes, which often leads to an exclusion of edges. This generalisation is useful if the spatial contribution of edges is low compared to homogenous regions. Then, the incorporation of edges, which are not strictly across track or along track, can lead to uncertainties in the estimation of striping magnitudes. However, if the spatial contribution of edges is relatively high, it may be not advisable to exclude them.

Exclusion would increase the uncertainties of any stripe assessment if the amount of remaining data is too low. To enable a robust decision, whether edges are incorporated or not, it is necessary to mask them beforehand.

A binary edge mask can be obtained for Remote Sensing Images (RSI) with wavelength-independent striping by applying the Hyperspectral Edge Detection Algorithm (HEDA) as proposed in [40], whereas the implemented Laplacian of Gaussian (LoG) filtering [41] should be only performed in striping direction to exclude striping edges. If wavelength-dependent striping or grey valued images must be destriped, another approach that adapts the Canny algorithm has been suggested [15]. This adaption comprises a substitution of the input image by the gradient of the input image in the direction of the stripes, which is the vertical along track direction. The Canny algorithm applied on a stripe-suppressed gradient image is then given an edge mask without stripe contributions. The basic Canny algorithm for a single banded image consists of multiple steps [15]:

1. Gradient estimation by convolving image with the derivative of a two-dimensional Gaussian.
2. Non-Maximum-Suppression of all edge pixels (edgels) which absolute gradient magnitude is lower than the magnitude of adjacent non edge pixels in perpendicular edge direction.
3. Hysteresis for all remaining edgels by tracing and thresholding edgels for given criteria.

To avoid uncertainties in choosing steering parameters of the Canny or HEDA algorithm and to minimise overestimations of across-track edges of heterogeneous regions, morphological dilations with small rectangular discs as structuring elements are performed [16,17]. The binary edge map (EM) that excludes striping is finally given, in short notation to:

(4) $$\text{EM}=({\underline{1}}_{\text{w}}⊗{\underline{1}}_{\text{w}})⊕\left\{\begin{array}{cc} \text{Canny}\;\left(\frac{\partial {\text{L}}_{\text{striped}}}{\partial r}\right), & \text{striping}=\text{striping}(\lambda )\\ \text{HEDA}({\text{L}}_{\text{striped}}), & \text{otherwise} \end{array}\right\}$$

where 1_w_ is a vector of width w valued 1, ⊗ is the dyadic product, ⊕ is morphological dilation, $\frac{\partial {\text{L}}_{\text{striped}}}{\partial \text{r}}$ is the gradient of the striped radiance image L_striped_ in striping (vertical) direction r and HEDA_striped_ is the HEDA adaption as given previously. The multiplicative application of the inverse edge map on L_striped_ then gives a HSI where high contrast edges are zeroed. This can be performed straightforwardly for each band as shown by the following equation:

(5) $${\text{L}}_{\text{striped},\;\text{flat}}={\left((1-{\text{EM}}^{\text{T}})⊗{\text{L}}_{\text{striped}}\right)}^{\text{T}}$$

To avoid uncertainties in the application of succeeding destriping approaches, masked or missing data as well as neighbour pixels are excluded. The workflow for masking of discontinuities of a hyperspectral image is then given by:

1. Computation of binary edge maps by the Hyperspectral Edge Detection Algorithm (HEDA) in striping direction (equation 4).
2. Morphological dilation to suppress edge related adjacency effects (e.g., PSF related blooming of edge spectra into homogeneous regions) as given by equation 4.
3. Binary filtering of the striped band by applying reciprocal binary edge map (equation 5).

### 3.3. Assessment of Slope c_1_ and Linear Reduction

If a scene constant, band and detector-element-dependent slope c_1_ is assumed, then c_1_ contributes to each element (pixel) within a column of one band the same multiplicative fraction (to avoid confusion in this work, the term ‘gain’ corresponds to the maximisation of the radiometric resolution [34]). The assessment of the c_1_ slope for each column and band can then be performed in multiple steps whereby a least squares polynomial fit is not required. In relation to equations (2) and (3) it follows that the difference of radiance data per detector and band is related only to c_1_ slope and to the difference of detected radiation equivalent DNs, because the offset c_0_ is constant per column and band and is eliminated in such a relation ( c1*DN1+ c0 – c1*DN2−c0 = c1*(DN1−DN2) ).

This basically reduces the mathematical complexity in the linear slope reduction case down to the retrieval and evaluation of the distribution of differences. Therefore, an elaborated polynomial fit is not necessary in the linear case. Because most miscalibrations can be corrected efficiently by linear reductions, the slope reductions are divided into a linear and a nonlinear case, whereby a potential multi-step approach for linear reduction is described in the following.

In the first step, the grey values or radiances for each band and column are sorted in ascending order. In the second step, spectrally unique values col(u) of a sorted and edge-map-filtered column vector col_s_ are extracted for each band by:

(6) $$\underline{\text{col}}(\underline{\text{u}})=\underline{\text{col}}\left({\text{row}}_{\text{s}}\underline{\left(\text{u}\right)}\right)\wedge \underline{\text{u}}=\text{diag}\left({\underline{\text{ind}}}_{\text{nr}}⊗{\left(\left[\begin{array}{c} {\underline{\text{col}}}_{{\text{s}}_{\left(2..\text{nr}\right)}}\\ {\text{col}}_{{\text{s}}_{1}} \end{array}\right]\neq {\underline{\text{col}}}_{\text{s}}\right)}^{T}\right)>0$$

where u denotes a unique index vector, nr denotes the number of rows, ind_nr_ denotes a column index vector ranging from 1 to nr of length nr, s denotes the sort index and $\left[\begin{array}{c} {\text{col}}_{{\text{s}}_{\left(2..\text{nr}\right)}}\\ {\text{col}}_{{\text{s}}_{1}} \end{array}\right]$ denotes col_s_ shifted backwards in place by one in row direction.

In a third step, the differences for each column of unique column values are used to detect the potential c_1_ slope of this column, which can be defined as:

(7) $${\underline{\text{diff}}}_{\text{c}1}({\underline{\text{u}}}_{\left(1..\text{nu}\right)},\lambda )=\underline{\text{col}}\;({\underline{\text{u}}}_{\left(2..\text{nu}\right)},\lambda )-\underline{\text{col}}\;({\underline{\text{u}}}_{\left(1..\text{nu}-1\right)},\lambda )$$

where nu is the number of elements of vector u. These column differences are then evaluated in a histogram in the fourth step. The minimum of the first bin (frequency category) of its normalised histogram P_1_(diff) always gives the smallest difference. This smallest difference is equivalent to c_1_ times the smallest difference of the unique values (SDUV) for each band and column of a not-striped representation of the striped image (perfectly calibrated). SDUV can be also interpreted as detector resolution. The resulting equation for the column-based estimation of the c_1_ slope is then given to:

(8) $${\text{c}}_{1}\approx \frac{\text{min}\left\{{\text{P}}_{1}({\underline{\text{diff}}}_{\text{c}1})\right\}}{\text{SDUV}}$$

If SDUV is one (e.g., often for grey valued images) then both sides of relation (8) are equivalent or else further processing necessary in which SDUV must be estimated.

Such an assessment can base on the median of column differences. This then gives the final relation for the estimation of c_1_ in short notation to:

(9) $${\text{c}}_{1}≅\frac{\text{min}\left\{{\text{P}}_{1}({\underline{\text{diff}}}_{{\text{c}}_{1}})\right\}}{\text{med}\left\{{\underline{\text{diff}}}_{{\text{c}}_{1}}\right\}}$$

In the fifth step, the application of the c_1_ slope reduction is verified. At first, the column c_1_ is compared to one to avoid unnecessary reductions. In this step, c_1_ is compared to c_1_ of the right adjacent column. This is performed by evaluating the difference in the related histograms for both columns. If the number of histogram bins and the positions of the maxima are equal for both columns, then c_1_ reduction should be not applied for that column, because it can be assumed that different c_1_ cause ‘stretches’ of the histogram and a shift between their maxima.

If these exclusion conditions are not fulfilled, then c_1_ is applied for that column by division. If there are also offset stripes, then these offsets are reduced concurrently to c_0_/c_1_, which can be minimised by a succeeding offset reduction. Subsequently, a rescaling of the data is required if offset stripes are present. Exclusion conditions and c_1_ reductions should be recorded concurrently to enable restoration of the original data, if cSNR indicates a necessary revoking of c_1_ reduction. Due to uncertainties in the assessment of SDUV, a linear radiometric rescaling should be performed after linear c_1_ reduction which is proposed in section 3.6. The respective workflow for the linear slope reduction per band is then given by:

1. Sorting of column radiances.
2. Extraction of unique column radiances (equation 6).
3. Calculation of differences of unique column differences (equation 7).
4. Estimation of slope per column as ratio of the smallest difference of unique values of a striped band (equation 8) and SDUV (equation 9).
5. Verification of the slope reduction necessity by evaluation of the shapes of the histograms of adjacent columns.

### 3.4. Assessment of Offset c_0_ and Linear Reduction

In the following, a multi-step approach for the reduction of offset miscalibrations is proposed that is conditioned by the assumptions that a radiometric offset is detector-element dependent and varies from scene to scene but not within a scene. This reduction approach consists of three steps that are applied consecutively. In the first step, two adjacent columns are considered. If these two columns cover a small, homogeneous area with assumed equal surface cover type, viewing geometry and second order effects [42], then the difference matrix diff_c0_ contains the offset difference which is given in the following to:

(10) $${\underline{\text{diff}}}_{\text{c}0}({\text{col}}_{2\ldots \text{nc}},\text{row},\lambda )={\text{L}}_{\text{striped},\;\text{flat}}({\text{col}}_{2\ldots \text{nc}},\text{row},\lambda )-L_{\text{striped},\;\text{flat}}({\text{col}}_{1\ldots \text{nc}-1},\text{row},\lambda )$$

where L_striped,flat_ denotes the striped hyperspectral image without edges (compare equation 5). The redundancy of the offset information is directly dependent on the number of small homogeneous regions with the same surface cover type and conditions as described beforehand as well as on the number of rows.

From this, it follows that remote sensing scenes may be especially convenient due to their along-track size. It also follows from this that a cluster agglomerating the majority of differences within the difference vector may most likely contain the offset reduction coefficient which is the basic assumption of this offset reduction approach. Thus, the distribution of the difference vector is examined in a normalised histogram in the second step. At first, the histogram is sorted in descending order according to its frequency as given in the following:

(11) $${\text{P}}_{\text{s}}(\text{L})=\text{P}{\left({\underline{\text{diff}}}_{\text{c}0}\right)}_{\text{s}}$$

Then, the number of the bin or the frequency category of the sorted histogram contributes to the rank of the probability of containing the offset reduction coefficient. In the third step, the first N bins of the differences histogram are considered, whereas N is user given and should be greater than or equal to 1. A representative offset reduction coefficient for each bin can then be obtained by computing the median value for each bin as given as follows:

(12) $${\text{c}}_{0}({\text{col}}_{2\ldots \text{nc}},\lambda )\approx \text{med}\left\{\text{med}\left\{{\text{P}}_{{\text{s}}_{\text{i}}}(\text{L})\right\}.\frac{n_{i}}{\Sigma_{i=1}^{N}n_{i}}\right\}\wedge \text{i}\in \text{N}$$

where n_i_ denotes the sorted frequency category i.

This concurrently reduces uncertainties caused by pre-processing such as the discontinuity masking. Subsequently, these bin reduction coefficients are weighted according to their frequencies. The median of the weighted bin correction factors is then supposed to be the offset correction coefficient of this column and is immediately subtracted. Coevally, the offset reduction coefficient is stored to rebuild the original data if succeeding cSNR estimation indicates a different stripe type. To reduce uncertainties in the determination of the offset deviation of the first column, a subsequent linear radiometric rescaling for each band is necessary, as is proposed in section 3.6. The workflow of the offset reduction per band can be summarised by:

1. Calculation of the differences of adjacent columns (equation 10).
2. Descending sorting of the estimated probability distributions of the column differences (equation 11).
3. Calculation of the average column difference per frequency category (equation 12).
4. Weighting of averaged column differences by their normalised frequencies.
5. Estimation of the column offset as average of all weighted average column differences in low SNR scenarios or as first weighted average column difference of a descending frequency sorted histogram of column differences in high SNR scenarious.

### 3.5. Assessment of Slopes c_2..M_ and Nonlinear Reduction

Nonlinear miscalibrations must incorporate correcting coefficients of higher degrees. It follows from this that additional repetitive information is crucial for the estimation of coefficients. Additionally, the distribution and scaling of the respective radiance domain is not known, which aggravates a polynomial radiance assignment. In the following, a multi-step band-wise approach is proposed, reducing both the influence of these limitations and nonlinear striping. It consists of a linear c_1_ and a nonlinear c_2..M_ reduction as well as a c_0_ reduction.

At first, the minimum and the maximum radiance per band as well as all unique column radiances per band are determined as described in Section 3.3. Secondly, the distribution of all column frequencies is considered. For this, a histogram can be used that is limited to the minimum and maximum radiance in this band and is binned with a frequency interval equal to the difference between maximum and minimum divided by the number of unique radiances of this band. This frequency interval then gives a domain for all unique column radiances for which it is assumed that dark current and saturation are similar for all detectors. Finally, unique column radiances are considered based on their frequencies in the previously defined histogram. The bin numbers or frequency interval numbers themselves then gives the domain per column, which could be considered to be quasi DN, and the unique column radiances can be fitted against their domain. For this purpose broadly used nonlinear least squares fits may be used. To avoid uncertainties in the determination of column offsets, the subsequent column-related reduction of nonlinearities by subtraction should be only applied on all column radiances with polynomial column coefficients greater than one. After this, a linear slope and offset correction should be applied, as previously described. Additionally, it is necessary to recover the radiance level and the radiance scaling of the entire band as described in Section 3.6. The respective workflow for the nonlinear reduction per band is given by:

1. Detection of minimum and maximum per band and extraction of unique column radiances (equation 6).
2. Definition of a histogram domain (x-axis) in respect to band maximum and minimum.
3. Extraction of frequency categories (x-vectors) per unique column radiances in respect to the previously defined histogram domain.
4. Least squares polynomial fit of column frequency categories (x-axis) and unique column radiances per column (y-axis).
5. Reduction of column nonlinearities by subtracting estimated column polynom c_2..M_.
6. Linear slope and offset reduction as given in Sections 3.3 and 3.4.

### 3.6. Scale Assessment

In order to reduce various effects of foregoing reductions a spectral rescaling is necessary. These effects can be caused by:

1. c_0_ offset reduction comprising offset relations.
2. linear c_1_ reduction potentially biased by differences between real and assessed SDUV.
3. nonlinear c_2..M_ reduction assuming similarities of band minima and dark currents as well as band maxima and saturations.

They can be minimised by rescaling the destriped radiance spectra to minimally striped areas. For this purpose a multistep approach was developed that detects and evaluates areas of lowest offset striping quantity. Based on these areas the whole band is rescaled by considering the spectral range before and after destriping.

At first, a striping quality indicator (SQI) is defined that combines both the level of stripes and their variation within a window. SQI can be defined as a vector of products of standard deviations of absolute reductions times the median of absolute reductions within a window of a pre-defined size (3 columns in size in minimum). Secondly, minima of SQI vectors are identified to detect minimal striped areas.

The position of the minimum is then indicating lowest reduction. The middle of the window is used as positional index for the column used as reference. To avoid rescaling of destriped images in reference to columns within this window, which are significantly miscalibrated, the reduction quantities should be considered. This can be performed by an evaluation of the ratio between the mean of first and last reduction coefficient within the minimum window and the reduction coefficient at the positional index. If all criteria are fulfilled, that is, a striping reduction was applied, SQI gives a positional index of minimal striped area, where striping is significant lower in the window, centred at the positional index, compared to other areas. Then, the last step—the scaling—is applied for each band at the positional index or column by:

(13) $${\text{L}}_{\text{rescaled}}=({\text{L}}_{\text{destriped}}-{\text{min}}_{\text{old}})\cdot \left(\frac{{\text{max}}_{\text{ref}}-{\text{min}}_{\text{ref}}}{{\text{max}}_{\text{old}}-{\text{min}}_{\text{old}}}\right)+{\text{min}}_{\text{ref}}$$

with min_old_ as local minima and max_old_ as local maxima in the destriped image window and min_ref_ as local minima and max_ref_ as local maxima in the striped image window similar to [34,43]. Hence, the work flow for the rescaling is then given by:

1. Computation of SQI within a moving window of a pre-defined size.
2. Definition of positional index by detecting the minimum within the SQI vector.
3. Rescaling of the whole band by applying equation 13 at the positional index.

### 3.7. Trend Reduction and Process Chain

In specific cases, brightness gradients of the destriped image can additionally disturb succeeding analyses. These trends may be caused by offset reduction undershoots or by material dependent effects due to varying illumination and acquisition geometry. In the following, a multi-step, band-related approach is proposed, which aims at the reduction of these effects. First, the median of each column is calculated, which is robust in the presence of outliers. This median vector v_med_ can be then boxcar convolved to get a smoothed vector representation vs of the columns means. This smoothed median vector vs indicates low frequency fractions of the column averages and, hence, potential undershoots or trends. Afterwards, the smoothed median vector can be mean normalised to disable reductions if there are no undershoots. To detrend the destriped image, the normalised, smoothed median vector can then be applied by division as given in:

(14) $${\text{L}}_{\text{detrend}}=\frac{{\text{L}}_{\text{rescaled}}}{{\underline{\text{ind}}}_{\text{nr}}⊗{\underline{\text{vs}}}_{\text{norm}}}\wedge \underline{\text{vs}}={\underline{\text{v}}}_{\text{med}}*\frac{{\underline{1}}_{\text{w}}}{\text{w}}$$

where vs_norm_ denotes the mean normalised smoothed median vector, v_med_ denotes the median vector, * denotes convolution and w the boxcar or window size. The workflow for detrending is then given by:

1. Calculation of the median vector consisting of the median values for each column.
2. Smoothing of the median vector to remove outliers.
3. Mean normalisation of the smoothed median vector to distinguish trends.
4. Detrending the rescaled, destriped band by applying Equation 14.

**Figure 4.**
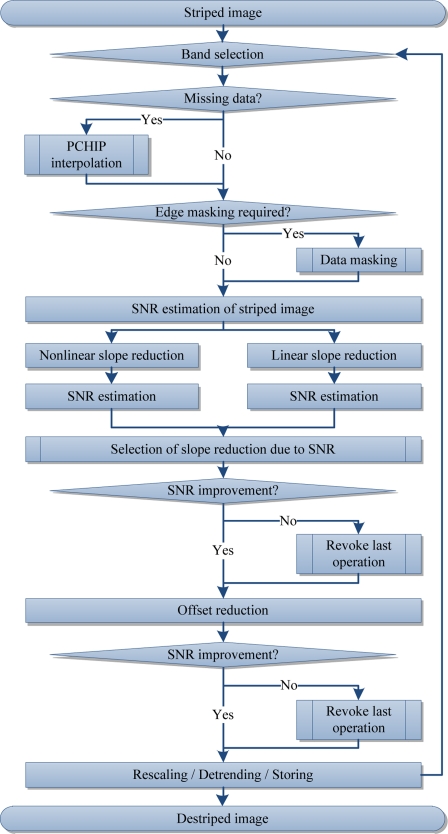
The destriping processing chain of the ‘ROM ’ framework.

Some of the sub steps of the previously proposed approach are limited to one spectral dimension. In consequence, it is recommended to destripe multi-dimensional data for each band as shown in Figure 4.

### 3.8. Evaluation Metrics

The global Peak-Signal-to-Noise-Ratio (PSNR) [40,44], the global Shannon Entropy [40,45] and the local Modified Structural Similarity Index (MSSIM) [4,44,46] were selected to objectively evaluate destriping outputs in comparison to striped inputs or ground truth. The PSNR considers the spectral ratio between band maximum and standard deviation, the Shannon Entropy incorporates spectral and spatial frequencies distributions and the MSSIM combines local structure, luminance and contrast metrics. All three image quality indicators were equally weighted.

## 4. Results and Discussion

Procedures to reduce radiometric miscalibration were automated to be repeatable.

### 4.1. Results for Destriping of Grey Valued Images

**Figure 5.**
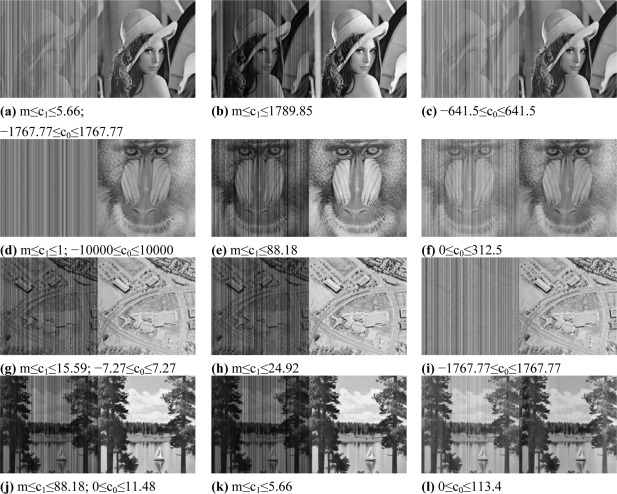
Exemplary striped grey scaled images (left) and destriping results (right) for slope c_1_ and offset c_0_ (a,d,g,j), slope c_1_ (b,e,h,k) and offset c_0_ reductions (c,f,i,l); m=0.0001.

The four grey valued images were selected to cover a broad range of spatial and spectral contributions. The ‘Lenna’ image provides a homogenous grey value distribution but contains also a long ‘natural’ vertical stripe in the left part of the image. The ‘Mandrill’ image is imbalanced due to spatial distribution of gradients and spectrally homogeneous regions. The ‘Aerial’ image has a homogenous grey value distribution, but lacks grey value variance. The ‘Sailboat on lake’ image can be characterised as having a balance of gradients and homogeneous regions as well as a homogeneous grey value distribution. Each grey valued image was differently striped 400 times and then destriped (see examples of Figure 5 for comparison) to assess the impact of different stripe types, of a priori knowledge and of specific image properties on the performance of the developed destriping approach.

All three image quality indicators—PSNR, Entropy and MSSIM [4,40,44–46]—were related to ground truth observations to avoid potential drawbacks that are associated with relying on a single type of evaluation approach [40], such as the universal image quality index [47].

Approximately 93% (100–7.3%) of the original information content was recovered (Table 1). The best results were achieved for the ‘Aerial’ image (∼98%) and for the ‘Sailboat on lake’ image (∼96%). Lowest recovery rates were obtained for destriping ‘Lenna’ (∼9 %) and ‘Mandrill’ (∼86%). C_1_ slope reduction outperformed offset reduction, whereby a priori knowledge had the highest impact on slope-related destriping. A visual inspection of all destriping results indicated that approximately 7% of them exhibited barely any detectable stripes that were potentially related to the information content quantity that could not be recovered. This supports the assumption that the combination of image quality indicators allows for the more efficient evaluation of image processing outputs [40]. Unbiased results were obtained for ‘Lenna,’ which diminished reductions in the area close to the long vertical structure (Figure 1a and 1c), as indicated by PSNR. The same applies to gradient-dominated images such as ‘Mandrill,’ in which the roughness-related entropy indicates the limitations of an adjacency-related c_0_-reduction approach (Figure 1d and f). The impact of the magnitude of miscalibration on the performance of proposed algorithm was also tested (Table 2).

According to the results presented in Table 2, the striping magnitude does not have a significant impact on the proposed reduction approach, indicating the robustness of the proposed approach.

On average, very high and robust image recoveries were achieved. This is exemplarily demonstrated for weak striping (e.g., Figure 5l) or for strong striping (e.g., Figure 5d). Images such as ‘Lenna’ with column-long, spectrally uniform structures or images such as ‘Mandrill,’ which are significantly dominated by edges, are not to be expected to be commonly observed by Remote Sensing. In summary, an overall recovery rate of 97% (the average for ‘Aerial’ and ‘Sailboat on lake’) was achieved—independent of linear stripe type and magnitude.

### 4.2. Results for Destriping of Hyperspectral AISA, Hyperion and EnMAP Scenes

The destriping results of the hyperspectral scenes were evaluated like the grey valued images. Nevertheless, two cases must be differentiated. Ground truth was available only for the EnMAP scene, and this scene was nonlinearly, artificially striped. The AISA and the Hyperion scenes were linearly destriped in association to the cSNR-related decision system.

The averages of image quality indicators are shown in Table 3, whereas only the rate of change could be computed for the AISA and the Hyperion scenes, because there was no ground truth information available. The AISA scenes were radiometrically recalibrated by approximately 5%. The cSNR decision system indicated that the AISA scenes were radiometrically miscalibrated due to dark current. Assuming that dark current does not change over time, the AISA striping must have been caused by offset miscalibration.

**Figure 6.**
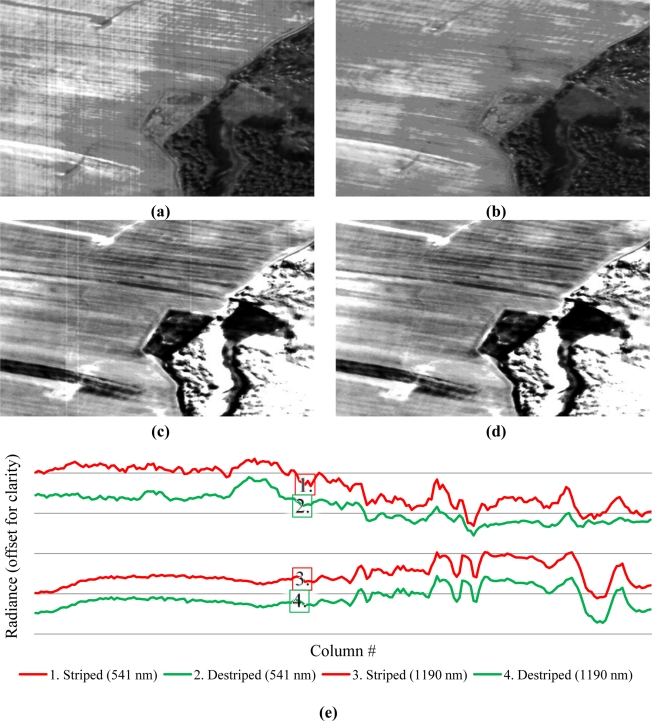
Exemplary grey scaled, striped images of sections in the middle of an AISA DUAL scene (VNIR-band 65–541 nm **(a)**, SWIR-band 283–1190 nm **(c)** and their respective radiometrically recalibrated results (VNIR **(b)**, SWIR **(d)** as well as a transect plot **(e)** through the middle of the same section for same VNIR and SWIR bands.

All bands of inspected AISA DUAL scenes showed miscalibrations (e.g., Figure 6). However, after destriping, miscalibration-related stripes appeared to be completely removed, resulting in an improvement of the MSSIM and the Entropy. The removed stripes were mostly non-negative, leading to a PSNR reduction because the band maxima must be lowered. A SNR of 20 was used as a general threshold for trend correction for each of the images. Consequently, a laboratory calibration of this AISA DUAL sensor is suggested.

As previously assumed, miscalibrations may vary only slowly over time. This indicates that corrections are highly correlated and that they show similar stripe patterns concurrently, which could be addressed by considering the gradients of removed stripes.

**Figure 7.**
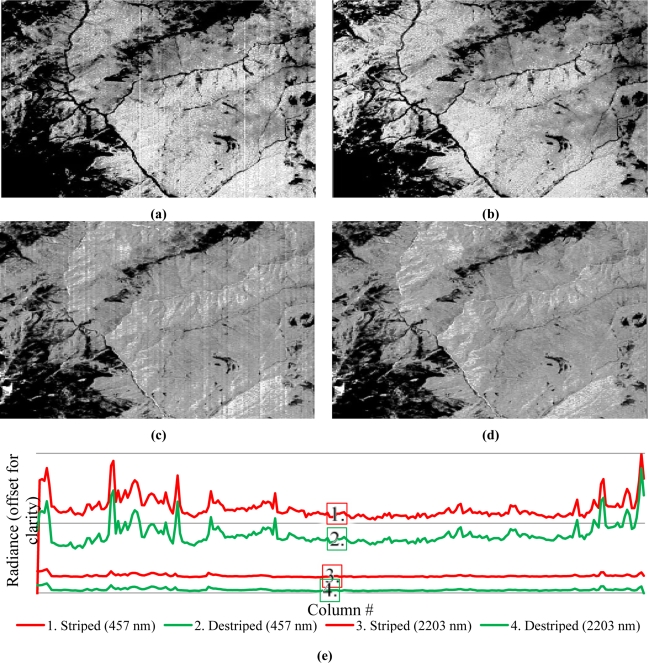
Exemplary grey scaled, striped images of sections in the middle of a Hyperion scene (VNIR-band 11–457 nm **(a)**, SWIR-band 205–2203 nm **(c)** and their respective radiometrically recalibrated results (VNIR **(b)**, SWIR **(d)** as well as a transect plot **(e)** through the middle of the same section for same VNIR and SWIR bands.

To enable a scene- and sensor-independent estimation of the stripe metrics of striped AISA and Hyperion data, the across-track gradients of the detected offset stripes were evaluated. At first, the gradients of the difference of the striped and the destriped images were computed for each band. Then, a random row of each gradient scene was selected. After this, the correlation coefficients between each scene and band were computed. Additionally, least squares regression coefficients for the correlation vector of each scene pair were estimated to detect potential trends. The average stripe correlations as well as the regression coefficients clearly indicate for the AISA DUAL and Hyperion scenes that the reductions are stable over time (Table 4). The Hyperion scenes were radiometrically recalibrated by approximately 4%. The cSNR decision system indicated that miscalibration is related to dark current. Similar to AISA, all miscalibration-related stripes appeared to be removed, as shown in Figure 7.

Missing values in the Hyperion scenes were interpolated by Piecewise Cubic Hermite Interpolation Polynomials in across- and along-track directions [4]. The average stripe correlation was higher when compared to AISA, which is considered unbiased for a satellite mounted sensor. However, for both types of scenes—AISA DUAL and Hyperion—in the SWIR spectral range, fewer offset miscalibrations were observed than were in the VNIR (as seen by comparison of Figures 6 a,b, 7 a,b and 6 c,d, 7 c,d, respectively). Due to the temporal stability of the obtained Hyperion offset recalibration dataset, it can be assumed that these offsets can be applied to other Hyperion scenes. Nevertheless, it is suggestible to rescale each AISA and Hyperion scene radiometrically to avoid any assumptions concerning the stability of a correction.

**Figure 8.**
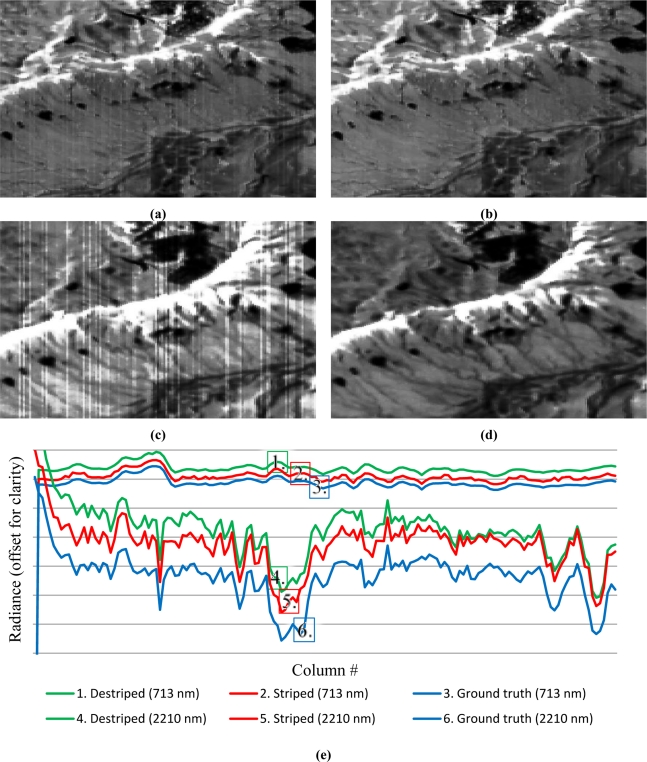
Exemplary grey scaled, striped images of sections in the middle of a EnMAP scene (VNIR-band 53–713 nm (**a**), SWIR-band 214–2210 nm (**c**) and their respective radiometrically recalibrated results (VNIR – (**b**), SWIR – (**d**)) as well as a transect plot (**e**) through the middle of the same section for same VNIR and SWIR bands.

In contrast to the AISA and Hyperion scenes, the EnMAP scene contains nonlinear, artificial effects and hence stripes appear (e.g., in Figure 8). Alike to the grey valued images (Figure 1) ground truth was available, which is required to evaluate the performance of the developed algorithm for the nonlinear case.

According to the results shown in Table 3 approximately 94% of the original scene quality could be recovered, similar to the results obtained for the grey valued sample images. Despite the lack of redundancy for the estimation of column-dependent calibration coefficients, this result demonstrates the broad applicability of the developed approach, whereas the results were completely automatically obtained without any scene-related ‘fine tuning’.

The striped representation of the EnMAP ground truth scene had an image quality deviation to ground truth of approximately 22%, that is, the overall image quality improvement between the striped and the striping reduced scene was approximately 21%. However, a subjective visual examination of the recalibrated EnMAP scenes has revealed that not all stripes were removed, corresponding to a detailed comparison between ground truth and destriped transects (Figure 8e). This is similar to the results for the grey scaled images, although different miscalibrations require different approaches to reduce them. This indicates that exclusive visual inspection of the AISA and Hyperion related destriping results leads to the incorrect assumption that all miscalibrations can completely be removed. According to the ground truth-related results for linearly striped grey valued sample images of similar grey value distributions, approximately 97% of a perfect calibration for the AISA and the Hyperion scenes have been achieved. Hence, a significant miscalibration reduction has been gained.

The proposed ROME framework is potentially affected by low SNR scenarios (SNR less than 20) where the offset reduction can cause frequency undershoots that are minimised by the proposed trend correction. However, sensitivity investigations have revealed that reduction quality is only weakly correlated to SNR.

Another aspect is the influence of natural, column-parallel, homogeneous regions covering the whole image extent on the offset reduction. Although the occurrence of such structures is not very likely for the majority of image data, they could be automatically detected and separately processed by repeated rescaling of these regions in order to avoid related artefacts.

## 5. Conclusions

The developed ROME framework for the reduction of linear and nonlinear miscalibration effects consists of two main parts—the spatial reduction of striping and the spectral rescaling of the image data. In order to reduce stripes and to preserve the spectral characteristics of observed surfaces both parts have to be executed consecutively.

The impact of these steps is proportional to the impact of the miscalibration on post-processing. Further processing steps, such as atmospheric correction and classification showed that uncorrected striped or miscalibrated data strongly affect subsequent analyses and result among others in striped columnar water vapour and aerosol optical thickness estimations and wrong segmentations. Hence, destriping is strongly recommended if data contain visually perceptible stripes after calibration.

The developed approach was widely tested and evaluated by different methodologies. In this process high calibration recovery rates of approximately 97% for linear miscalibration and approximately 94% for nonlinear miscalibration have been achieved. It was shown that linear and nonlinear miscalibration-related striping can be efficiently suppressed without a significant loss of radiometric scaling and variance information or gradient magnitudes information. Furthermore, destriping did not lead to artefacts in the resulting image spectra which is exemplary demonstrated by the profiles shown in Figures 6–8e.

Concurrently, the successful destriping of images of different origin, stripe type and magnitude demonstrates the broad applicability, robustness and the high performance of the developed approach.

Additionally, the full automation, the reduced mathematical complexity of the proposed method, as well as the insensitivity to *a priori* knowledge, indicate operational capabilities. Nevertheless, there is still place for improvements, for example, through an information-related redundancy amplification for the assessment of nonlinear calibration coefficients.

## Figures and Tables

**Table 1.** Average deviation of the 1600 destriped images from ground truth [%].

**Stripe type**	Flag^*^	**PSNR**				**Entropy**				**MSSIM**				**Average**
		**Im 1^1^**	**Im 2^2^**	**Im 3^3^**	**Im 4^4^**	**Im 1^1^**	**Im 2^2^**	**Im 3^3^**	**Im 4^4^**	**Im 1^1^**	**Im 2^2^**	**Im 3^3^**	**Im 4^4^**	

**ALL**		16.4	13.0	1.6	4.2	7.5	29.6	2.8	2.7	9.6	9.0	0.7	2.8	**8.3**

**Offset**	o	15.2	13.0	4.7	10.6	7.0	29.6	8.4	5.1	9.0	9.0	2.2	8.6	**10.2**
	x	16.8	13.0	5.1	14.5	7.7	29.5	9.2	7.1	9.7	9.0	1.9	8.1	**11.0**

**Slope**	o	16.8	13.0	0.0	0.0	7.7	29.6	0.0	0.0	9.7	9.0	0.0	0.0	**7.1**
	x	0.0	0.0	0.0	0.0	0.0	0.0	0.0	0.0	0.0	0.0	0.0	0.0	**0.0**

**Average**		**13.1**	**10.4**	**2.3**	**5.9**	**6.0**	**23.6**	**4.1**	**3.0**	**7.6**	**7.2**	**1.0**	**3.9**	**7.3**

Flag denotes by ‘o’ no and by ‘x’ a-priori knowledge of the stripe type;

1 Im 1 is the ‘Lenna’ image;

2 Im 2 is the ‘Mandrill’ image;

3 Im 3 is the ‘Aerial’ image;

4 Im 4 is the ‘Sailboat on lake’ image.

**Table 2.** Average stripe magnitude impact on this approach for all destriped images [%].

**Stripe Type**	Flag*	**PSNR**				**Entropy**				**MSSIM**				**Average**
		Im 1^1^	Im 2^2^	Im 3^3^	Im 4^4^	Im 1^1^	Im 2^2^	Im 3^3^	Im 4^4^	Im 1^1^	Im 2^2^	Im 3^3^	Im 4^4^	

**ALL**		0.8	0.0	2.1	6.2	0.8	0.0	2.1	6.2	0.4	0.0	3.8	4.7	**2.3**

**Offset**	o	1.7	0.0	3.1	7.7	1.7	0.0	3.1	7.7	0.9	0.0	5.9	5.7	**3.1**
	x	0.4	0.0	3.4	9.8	0.4	0.0	3.4	9.8	0.2	0.0	6.5	7.2	**3.4**

**Slope**	o	0.4	0.0	1.6	4.0	0.4	0.0	1.6	4.0	0.2	0.0	2.9	2.8	**1.5**
	x	14.1	11.5	1.6	4.0	14.1	11.5	1.6	4.0	8.2	42.0	2.9	2.8	**9.8**

**Average**		**3.6**	**2.4**	**2.4**	**6.4**	**3.6**	**2.4**	**2.4**	**6.4**	**5.0**	**25.1**	**3.6**	**4.5**	**5.6**

Flag denotes by ‘o’ no and by ‘x’ a priori knowledge of the stripe type;

1 Im 1 is the ‘Lenna’ image;

2 Im 2 is the ‘Mandrill’ image;

3 Im 3 is the ‘Aerial’ image;

4 Im 4 is the ‘Sailboat on lake’ image.

**Table 3.** Image quality indices for destriped hyperspectral scenes as rate of change [%].

**Sensor**	**Scene**	**PSNR**	**Entropy**	**MSSIM**	**Average**

**AISA**	Scene 1	−0.8	5.0	9.2	**4.4**
	Scene 2	−2.1	10.1	8.3	**5.5**
	Scene 3	−1.6	8.6	7.7	**4.9**

	**Average**	**−1.5**	**7.9**	**8.4**	**4.9**

**Hyperion**	Scene 1	1.2	3.1	6.0	**3.4**
	Scene 2	1.5	5.1	6.8	**4.5**

	**Average**	**1.4**	**4.1**	**6.4**	**4.0**

**EnMAP**	Scene 1	**2.4**	**8.0**	**6.5**	**5.6**

**Table 4.** Inter-scene striping relations.

**Sensor**	**Scene**	**Slope**	**Offset**	**Average stripe correlation R^2^**

**AISA**	Scene 1 to Scene 2	−0.0013	0.84	**0.85**
	Scene 1 to Scene 3	−0.0021	0.93	
	Scene 2 to Scene 3	−0.0012	0.85	

**Hyperion**	Scene 1 to Scene 2	−0.0001	0.92	**0.92**

## References

[1] Datt B, McVicar TR, van Niel TG, Jupp DLB, Pearlman JS (2003). Preprocessing EO-1 Hyperion Hyperspectral Data to Support the Application of Agricultural Indexes. IEEE Trans. Geosci. Rem. Sens.

[2] Oppelt N, Mauser W (2007). The Airborne Visible/Infrared Imaging Spectrometer Avis: Design, Characterization and Calibration. Sensors.

[3] Oliveira P, Gomes L (2010). Interpolation of Signals with Missing Data Using Principal Component Analysis. Multidimens. Syst. Signal Process.

[4] Tsai F, Chen W (2008). Striping Noise Detection and Correction of Remote Sensing Images. IEEE Trans. Geosci. Rem. Sens.

[5] Cavalli R, Fusilli L, Pascucci S, Pignatti S, Santini F (2008). Hyperspectral Sensor Data Capability for Retrieving Complex Urban Land Cover in Comparison with Multispectral Data: Venice City Case Study (Italy). Sensors.

[6] Le Maire G, François C, Soudani K, Berveiller D, Pontailler J-Y, Bréda N, Genet H, Davi H, Dufrêne E (2008). Calibration and Validation of Hyperspectral Indices for the Estimation of Broadleaved Forest Leaf Chlorophyll Content, Leaf Mass Per Area, Leaf Area Index and Leaf Canopy Biomass. Rem. Sens. Environ.

[7] Liu B, Zhang L, Zhang X, Zhang B, Tong Q (2009). Simulation of EO-1 Hyperion Data from ALI Multispectral Data Based on the Spectral Reconstruction Approach. Sensors.

[8] García J, Moreno J Removal of Noises in CHRIS/Proba Images: Application to the SPARC Campaign Data.

[9] Shen HF, Ai TH, Li PX Destriping and Inpainting of Remote Sensing Images Using Maximum a-Posteriori Method.

[10] Simpson JJ, Gobat JI, Frouin R (1995). Improved Destriping of Goes Images Using Finite Impulse Response Filters. Rem. Sens. Environ.

[11] Simpson JJ, Stitt JR, Leath DM (1998). Improved Finite Impulse Response Filters for Enhanced Destriping of Geostationary Satellite Data. Rem. Sens. Environ.

[12] Bouali M, Ladjal S A Variational Approach for the Destriping of Modis Data.

[13] Carfantan H, Idier J (2010). Statistical linear destriping of satellite-based pushbroom-type images. IEEE Trans. Geosci. Rem. Sens.

[14] Gómez-Chova L, Alonso L, Guanter L, Camps-Valls G, Calpe J, Moreno J (2008). Correction of Systematic Spatial Noise in Push-Broom Hyperspectral Sensors: Application to CHRIS/Proba Images. Appl. Opt.

[15] Canny J (1986). A Computational Approach to Edge Detection. IEEE Trans. Pattern Anal. Mach. Intell.

[16] Haralick RM, Sternberg SR, Zhuang X (1987). Image Analysis Using Mathematical Morphology. IEEE Trans. Pattern Anal. Mach. Intell.

[17] Rogass C, Itzerott S, Schneider B, Kaufmann H, Hüttl R (2009). Edge Segmentation by Alternating Vector Field Convolution Snakes. Int. J. Comput. Sci. Netw. Secur.

[18] Guanter L, Segl K, Kaufmann H (2009). Simulation of optical remote-sensing scenes with application to the enmap hyperspectral mission. IEEE Trans. Geosci. Rem. Sens.

[19] Kaufmann H, Segl K, Guanter L, Förster KP, Stuffler T, Müller A, Richter R, Bach H, Hostert P, Chlebek C Environmental Mapping and Analysis Program (EnMAP)—Recent Advances and Status.

[20] Segl K, Guanter L, Kaufmann H, Schubert J, Kaiser S, Sang B, Hofer S (2010). Simulation of Spatial Sensor Characteristics in the Context of the EnMAP Hyperspectral Mission. IEEE Trans. Geosci. Rem. Sens.

[21] Spectral Imaging Ltd Aisa Dual, 2nd Version. http://www.specim.fi/media/aisa-datasheets/dual_datasheet_ver2-10.pdf.

[22] Pearlman JS, Barry PS, Segal CC, Shepanski J, Beiso D, Carman SL (2003). Hyperion, a Space-Based Imaging Spectrometer. IEEE Tran. Geosci. Rem. Sens.

[23] Ungar SG, Pearlman JS, Mendenhall JA, Reuter D (2003). Overview of the Earth Observing One (EO-1) Mission. IEEE Trans. Geosci. Rem. Sens.

[24] Weber A (1997). The USC-SIPI Image Database.

[25] Box G, Muller M (1958). A Note on the Generation of Random Normal Deviates. Ann. Math. Stat.

[26] Roessner S, Wetzel H-U, Kaufmann H, Sarnagoev A (2005). Potential of Satellite Remote Sensing and GIS for Landslide Hazard Assessment in Southern Kyrgyzstan (Central Asia). Nat. Hazards.

[27] Gersman R, Ben-Dor E, Beyth M, Avigad D, Abraha M, Kibreab A (2008). Mapping of Hydrothermally Altered Rocks by the EO-1 Hyperion Sensor, Northern Danakil Depression, Eritrea. Int. J. Rem. Sens.

[28] Kruse F, Boardman J, Huntington J (2003). Comparison of Airborne Hyperspectral Data and EO-1 Hyperion for Mineral Mapping. IEEE Trans. Geosci. Rem. Sens.

[29] Biggar S, Thome K, Wisniewski W (2003). Vicarious Radiometric Calibration of EO-1 Sensors by Reference to High-Reflectance Ground Targets. IEEE Trans. Geosci. Rem. Sens.

[30] Bruegge C, Diner D, Kahn R, Chrien N, Helmlinger M, Gaitley B, Abdou W (2007). The Misr Radiometric Calibration Process. Rem. Sens. Environ.

[31] Bindschadler R, Choi H (2003). Characterizing and Correcting Hyperion Detectors Using Ice-Sheet Images. IEEE Trans. Geosci. Rem. Sens.

[32] Dell’Endice F Improving the Performance of Hyperspectral Pushbroom Imaging Spectrometers for Specific Science Applications.

[33] Dell'Endice F, Nieke J, Koetz B, Schaepman ME, Itten K (2009). Improving Radiometry of Imaging Spectrometers by Using Programmable Spectral Regions of Interest. ISPRS J. Photogramm. Rem. Sens.

[34] Chander G, Markham B, Helder D (2009). Summary of Current Radiometric Calibration Coefficients for Landsat MSS, TM, ETM+, and EO-1 ALI Sensors. Rem. Sens. Environ.

[35] Barducci A, Castagnoli F, Guzzi D, Marcoionni P, Pippi I, Poggesi M (2004). Solar Spectral Irradiometer for Validation of Remotely Sensed Hyperspectral Data. Appl. Opt.

[36] Xiong X, Barnes W (2006). An Overview of Modis Radiometric Calibration and Characterization. Adv. Atmos. Sci.

[37] Brunn A, Fischer C, Dittmann C, Richter R Quality Assessment, Atmospheric and Geometric Correction of Airborne Hyperspectral Hymap Data.

[38] Gao B-C (1993). An Operational Method for Estimating Signal to Noise Ratios from Data Acquired with Imaging Spectrometers. Rem. Sens. Environ.

[39] Atkinson PM, Sargent IM, Foody GM, Williams J (2005). Interpreting Image-Based Methods for Estimating the Signal-to-Noise Ratio. Int. J. Rem. Sens.

[40] Rogass C, Itzerott S, Schneider B, Kaufmann H, Hüttl R (2010). Hyperspectral Boundary Detection Based on the Busyness Multiple Correlation Edge Detector and Alternating Vector Field Convolution Snakes. ISPRS J. Photogramm. Rem. Sens.

[41] Marr D, Hildreth E (1980). Theory of Edge Detection. Proc. Roy. Soc. Lond. B. Biol. Sci.

[42] Richter R (1997). Correction of Atmospheric and Topographic Effects for High Spatial Resolution Satellite Imagery. Int. J. Rem. Sens.

[43] Schroeder T, Cohen W, Song C, Canty M, Yang Z (2006). Radiometric Correction of Multi-Temporal Landsat Data for Characterization of Early Successional Forest Patterns in Western Oregon. Remote Sens. Environ.

[44] Wang Z, Bovik AC (2009). Mean Squared Error: Love It or Leave It? A New Look at Signal Fidelity Measures. IEEE Signal Process. Mag.

[45] Frank S, Smith E (2010). Measurement Invariance, Entropy, and Probability. Entropy.

[46] Wang Z, Bovik AC, Sheikh HR, Simoncelli EP (2004). Image Quality Assessment: From Error Visibility to Structural Similarity. IEEE Trans. Image Process.

[47] Wang Z, Bovik AC (2002). A Universal Image Quality Index. IEEE Signal Process. Lett.

